# Ubiquitin and Ubiquitin-Like Proteins Are Essential Regulators of DNA Damage Bypass

**DOI:** 10.3390/cancers12102848

**Published:** 2020-10-02

**Authors:** Nicole A. Wilkinson, Katherine S. Mnuskin, Nicholas W. Ashton, Roger Woodgate

**Affiliations:** Laboratory of Genomic Integrity, National Institute of Child Health and Human Development, National Institutes of Health, 9800 Medical Center Drive, Rockville, MD 20850, USA; nicole.wilkinson@nih.gov (N.A.W.); kmnuskin@osteo.wvsom.edu (K.S.M.)

**Keywords:** mutagenesis, carcinogenesis, DNA damage bypass, DNA damage tolerance, translesion synthesis, template switching, ubiquitination, SUMOylation, NEDDylation, ISGylation

## Abstract

**Simple Summary:**

Ubiquitin and ubiquitin-like proteins are conjugated to many other proteins within the cell, to regulate their stability, localization, and activity. These modifications are essential for normal cellular function and the disruption of these processes contributes to numerous cancer types. In this review, we discuss how ubiquitin and ubiquitin-like proteins regulate the specialized replication pathways of DNA damage bypass, as well as how the disruption of these processes can contribute to cancer development. We also discuss how cancer cell survival relies on DNA damage bypass, and how targeting the regulation of these pathways by ubiquitin and ubiquitin-like proteins might be an effective strategy in anti-cancer therapies.

**Abstract:**

Many endogenous and exogenous factors can induce genomic instability in human cells, in the form of DNA damage and mutations, that predispose them to cancer development. Normal cells rely on DNA damage bypass pathways such as translesion synthesis (TLS) and template switching (TS) to replicate past lesions that might otherwise result in prolonged replication stress and lethal double-strand breaks (DSBs). However, due to the lower fidelity of the specialized polymerases involved in TLS, the activation and suppression of these pathways must be tightly regulated by post-translational modifications such as ubiquitination in order to limit the risk of mutagenesis. Many cancer cells rely on the deregulation of DNA damage bypass to promote carcinogenesis and tumor formation, often giving them heightened resistance to DNA damage from chemotherapeutic agents. In this review, we discuss the key functions of ubiquitin and ubiquitin-like proteins in regulating DNA damage bypass in human cells, and highlight ways in which these processes are both deregulated in cancer progression and might be targeted in cancer therapy.

## 1. Introduction

Carcinogenesis is a multi-step process predominantly driven by DNA mutations. These driver mutations provide cancer cells with a selective growth advantage, facilitating tumorigenesis and the eventual progression to malignancy. Mistakes during DNA replication, as well as during the repair of damaged DNA, can result in DNA mutations [1]. DNA can be damaged when cells are exposed to chemical mutagens, such as carcinogenic nitroaromatics and aflatoxins, as well as physical agents like ultraviolet (UV) radiation [1]. In addition, DNA is constantly damaged by free radicals and other metabolic byproducts that result from normal cellular metabolism [2]. In cancers, the rate at which DNA damage causes mutations is often accelerated by the somatic or acquired mutation of DNA replication and repair proteins.

One of the ways DNA damage causes mutations is by acting as a physical barrier to replication. The resulting ‘replication stress’ can lead to a state of genomic instability and is a hallmark of many pre-cancerous and cancerous cells [3,4]. DNA damage bypass is one approach cells use to prevent the replication stress caused by persistent DNA lesions and can be accomplished by three pathways: (1) translesion DNA synthesis (TLS), (2) template switching (TS) and (3) repriming by the primase-polymerase, PrimPol. While mechanistically dissimilar, each pathway allows replication to bypass DNA lesions, leaving the damage to be repaired at a later time.

Ubiquitin and ubiquitin-like proteins are essential post-translational modifiers that regulate the stability, localization, and activity of proteins that function in a diverse range of cellular processes. The important roles of these modifiers are highlighted by the identification of many cancers, resulting from the deregulated metabolism of ubiquitin and ubiquitin-like proteins. The prevalence of such cancers has indeed been increasingly discussed in recent years [5,6,7]. In this review, we provide a unique perspective, where we discuss the essential roles of these modifiers in regulating DNA damage bypass, as well as how the disruption of these processes can contribute to cancer development. In addition, we discuss how targeting these processes may be an effective strategy in cancer therapy.

## 2. Ubiquitin and Ubiquitin-Like Modifiers

Ubiquitin is a highly conserved 76 amino acid protein expressed by all eukaryotic organisms [8]. The addition of ubiquitin to a target protein is predominantly catalyzed by a three-enzyme cascade [9]. In the first step, an E1 ubiquitin activating enzyme binds to ubiquitin and adenosine triphosphate (ATP), and catalyzes adenylation of the ubiquitin C-terminal acyl group. The E1 catalytic cysteine residue then attacks the ubiquitin adenylate, displacing the adenosine monophosphate (AMP) molecule to form a thioester-linked E1-ubiquitin conjugate [10]. In the second step, the ubiquitin moiety is then transferred to an E2 conjugating enzyme via a transthiolation reaction [11]. The third step is then catalyzed by an E3 ubiquitin ligase, which binds to and facilitates ubiquitination of a substrate protein. This results in formation of an isopeptide bond between the ubiquitin C-terminal glycine (G76) and the ε-amino group of a substrate lysine residue [12]. Although E3 ubiquitin ligases have an important role in substrate recognition and facilitate most ubiquitination reactions, as we will discuss in Section 4.5, some instances of E3-independent ubiquitination have also been described involving ubiquitin-binding substrates [13]. In addition, an instance of E1 and E2-independent ubiquitination was recently described, mediated by the sidE ubiquitinating enzyme of *Legionella pneumophila* [14]. For the most part, however, the three-enzyme cascade remains a central tenet of ubiquitin conjugation. Ubiquitination is also further regulated by the opposing function of deubiquitination enzymes (DUBs), a class of specialized proteases that catalyze the deconjugation of ubiquitin from a modified protein [15].

**Figure 1:**
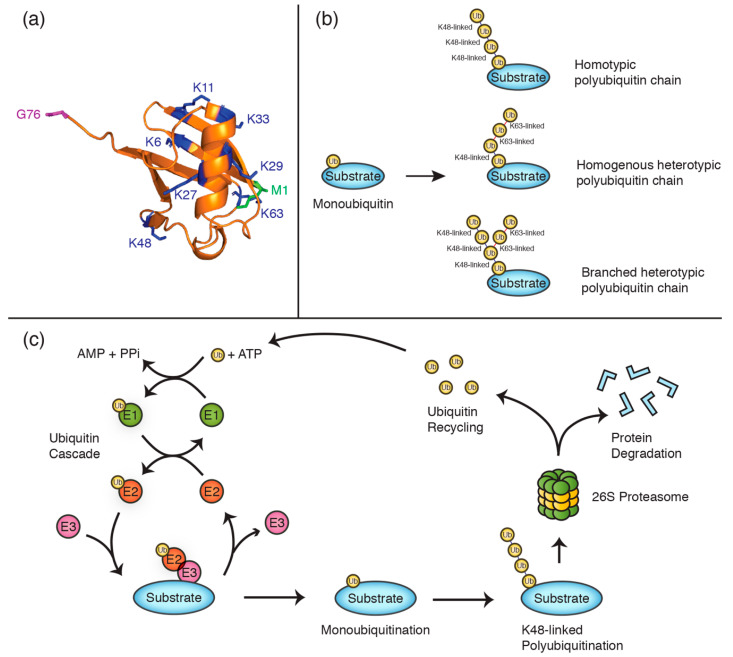
Polyubiquitin chains are constituted by specific lysine linkages. (**a**) Cartoon structure of ubiquitin (PDB 1ubq) with lysine residues illustrated in blue, the N-terminal methionine (M1) in green, and the C-terminal glycine residue (G76) in purple. (**b**) Schematics exemplifying different polyubiquitin chain types. (**c**) Schematic of the ubiquitination cycle, illustrating the proteolytic degradation of a K48-linked polyubiquitinated substrate and ubiquitin recycling.

Although some protein substrates are modified by monoubiquitination, many others are instead regulated by polyubiquitination, where one of the seven lysine residues on ubiquitin, or the N-terminal methionine residue, is further modified to form ubiquitin chains (Figure 1a–c). Ubiquitin chains can be either homotypic, where ubiquitin moieties are all linked via the same residues (e.g., K48), or heterotypic, where different linkage types occur within the same chain (e.g., a combination of K48 and K63 linkages). In addition, chains can be either homogenous, where each ubiquitin is further ubiquitinated by only one other ubiquitin moiety, or branched, where ubiquitin groups within the chain are ubiquitinated on more than one lysine residue. The effect of these ubiquitin chains on the substrate protein varies depending on the lysine linkages used [16]. For instance, while K48-linked chains typically target proteins for degradation by the proteasome [17], other linkages mostly regulate non-proteolytic functions such as protein trafficking [16,18]. The diverse functionality of ubiquitin modifications is largely dictated by specific interactions with proteins containing ubiquitin-binding domains (UBDs) [17,19]. The adaptor proteins Rad23A and Rad23B, for instance, preferentially bind K48-linked polyubiquitin chains and shuttle substrate proteins to the proteasome for degradation [20]. In DNA damage bypass, mono- and polyubiquitination also plays an important non-proteolytic role in recruiting ubiquitin-binding proteins to sites of replication.

**Figure 2:**
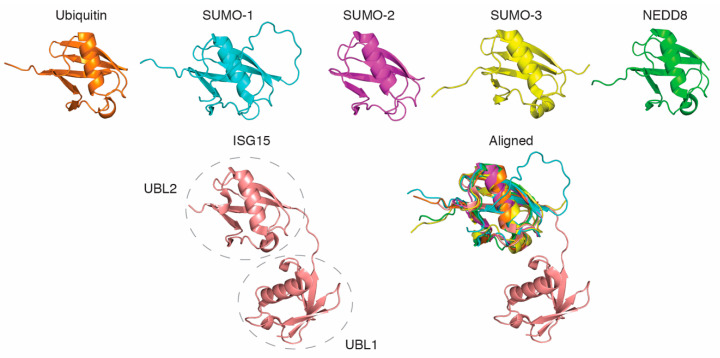
All ubiquitin-like proteins contain one or more structurally near-identical ubiquitin-like folds. The figure represents the individual and aligned structures of ubiquitin (PDB 1ubq), NEDD8 (PDB 2ko3), SUMO-1 (PDB 2n1v), SUMO-2 (PDB 1WM3), SUMO-3 (PDB 1u4a), and ISG15 (PDB 1Z2M). The dashed circles indicate the two ubiquitin-like domains (UBL1 and UBL2) of ISG15.

Aside from ubiquitin, a number of other small ubiquitin-like proteins also have essential roles as post-translational regulators of DNA damage bypass [21]. These proteins include the SUMO (small ubiquitin-like modifier) protein family (SUMO-1, 2, and 3), NEDD8 (neuronal precursor cell-expressed developmentally down-regulated protein 8), and ISG15 (interferon-stimulated gene 15). While these proteins share only limited primary sequence similarity with ubiquitin, each protein forms one or two structurally near-identical ubiquitin-like fold(s) (Figure 2). Each of these modifiers can also be conjugated to substrate proteins via three-enzyme reactions analogous to ubiquitination, where they mediate a range of (mostly) non-proteolytic functions.

## 3. DNA Damage Bypass

In eukaryotic cells, DNA is predominantly replicated by the three B-family DNA polymerases, Pol α (alpha), Pol δ (delta), and Pol ε (epsilon) [22]. Each of these ‘replicative’ polymerases feature highly selective active sites that enable them to synthesize new DNA with high fidelity [23]. However, these restrictive active sites also prevent these polymerases from replicating damaged templates, causing them to stall upstream of DNA lesions [24]. These barriers must be overcome to ensure the complete duplication of the DNA prior to cell division, as well as to prevent formation of lethal double-strand breaks (DSBs) due to the collapse of replication forks following their prolonged stalling [25].

**Figure 3:**
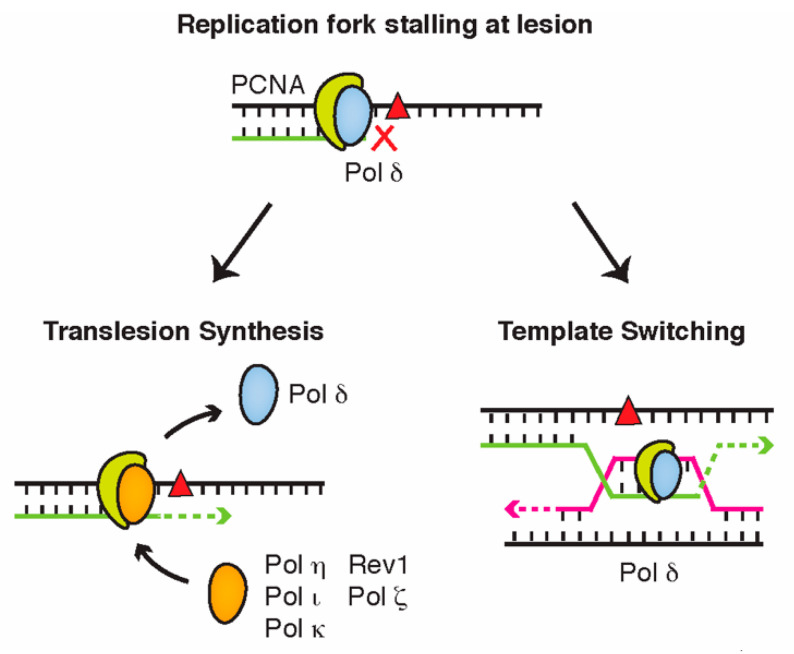
DNA damage bypass allows cells to evade lesions that stall replicative polymerases. During translesion synthesis, the replicative polymerase is replaced by a specialized translesion polymerase that can replicate past the lesion. In template switching, the replisome bypasses the lesion by temporarily replicating the nascent strand of a sister chromatid.

Translesion synthesis (TLS) and template switching (TS) are two pathways that cells use to relieve the replication stress caused by persistent DNA lesions. During TLS, the stalled replicative polymerase is temporarily replaced by a specialized translesion polymerase, which is able to incorporate nucleotides across from bulky lesions due to their open and flexible active sites [26] (Figure 3). Eukaryotic TLS polymerases include Pol η (eta), Pol ι (iota), Pol κ (kappa), and Rev1 within the Y-family, as well as Pol ζ (zeta) of the B-family DNA polymerases. Although each TLS polymerase can bypass a range of different lesions, it is generally believed that each is specialized to accurately bypass a specific type of DNA damage, referred to as their ‘cognate lesion’ [27]. Pol η, for instance, has an important role in accurately bypassing thymidine dimers caused by ultraviolet light [28,29], while Pol κ is mainly used to replicate passed DNA lesions on the N^2^ position of guanine [30,31].

While TLS alleviates much of the genome instability risk caused by replication stalling, it comes at the cost of lower replication fidelity—a consequence of the more accommodating active sites of TLS polymerases—and an increased risk of mutagenesis [32]. By contrast, TS is considered an error-free mechanism of DNA damage bypass as the synthesis of new nucleotides is still mediated by standard replicative polymerases. In this pathway, rather than switching polymerases, the stalled replisome instead switches templates and bypasses the lesion by replicating the newly synthesized nascent strand of its sister chromatid (Figure 3). This occurs via a Rad51-dependent strand invasion mechanism, akin to that employed during homologous recombination (HR) [33].

A third mechanism of DNA damage bypass has also recently been suggested, mediated by the primase-polymerase, PrimPol. Although PrimPol possesses some TLS-like activity, its main role in damage bypass seems to come from its ability to reprime and restart replication downstream of a DNA damage lesion [34]. Unlike TLS and TS, which are both intricately regulated by ubiquitin and ubiquitin-like proteins, no such regulatory network has been described to date for DNA damage bypass by PrimPol repriming. We will therefore primarily focus on TLS and TS for the remainder of this review, as we discuss the roles of ubiquitin and ubiquitin-like proteins in regulating DNA damage bypass.

While our discussion above illustrates DNA damage bypass as a co-replicative process that occurs in concert with an ongoing replication fork, there is evidence that each mode of bypass can also occur post-replicatively, during late S and G2 of the cell cycle. In this scenario, TLS and TS are thought to function in sealing ssDNA gaps that arise when a stalled replisome is disassembled and reassembled downstream of a lesion, leaving an unreplicated stretch behind it [35]. Such post-replicative repair is essential to ensure the completion of DNA replication prior to cell division.

## 4. Ubiquitin and Ubiquitin-Like Modifiers in DNA Damage Bypass

### 4.1. PCNA Ubiquitination Is a Central Regulator of Translesion Synthesis and Template Switching

Ubiquitin is added to and removed from many proteins directly involved in DNA damage bypass. The proliferating cell nuclear antigen (PCNA) sliding clamp is one such substrate that has essential roles in normal replication as well as in TLS and TS [36]. Eukaryotic PCNA functions as a heterotrimeric protein, forming a ring-like structure through which dsDNA at the replication fork is encircled [37]. During normal DNA replication, Pol δ associates with PCNA through its PCNA-Interacting Protein (PIP) motifs to form a holoenzyme that replicates the lagging strand [38]. In a similar way, TLS polymerases also associate with PCNA through PIP box motifs. Unlike the conserved PIP box of Pol δ and other PCNA-binding proteins, however, most TLS polymerases contain ‘non-canonical’ PIP box sequences which mediate a comparatively transient interaction with PCNA [39]. This effectively limits the ability of TLS polymerases to compete with Pol δ for access to PCNA during normal DNA replication.

**Figure 4:**
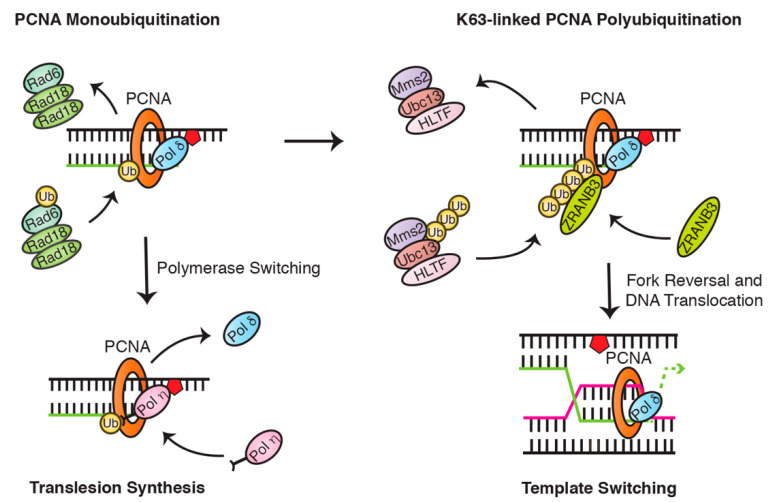
Mono- and polyubiquitination of proliferating cell nuclear antigen (PCNA) tightly regulates the initiation of translesion synthesis and template switching. In humans, PCNA is monoubiquitinated by the Rad6/Rad18 complex and can be subsequently polyubiquitinated by the Ubc13/Mms2/HLTF complex.

The monoubiquitination of PCNA is an important step in the exchange between Pol δ and TLS polymerases [32] (Figure 4). Following replication fork stalling, the accumulation of replication protein A (RPA) acts to recruit the Rad6 (E2)/Rad18 (E3) complex to sites of damage, which sequentially transfers a single ubiquitin moiety to PCNA lysine residue 164 (K164) [40,41]. This modification forms a binding platform for Y-family TLS polymerases, which contain ubiquitin-binding domains (UBDs) in their C-terminal regions. These domains can be one of two types: ubiquitin-binding zinc finger domains (UBZs) or ubiquitin-binding motifs (UBMs). While Rev1 and Pol ι both have two UBMs, Pol η and Pol κ have one or two UBZs, respectively [27]. Although there has been some debate as to whether PCNA monoubiquitination is strictly essential for initiating translesion synthesis [42], numerous groups have nevertheless demonstrated that PCNA monoubiquitination strongly promotes TLS polymerase recruitment to the replication fork by providing an additional means through which these polymerases can compete for PCNA binding [43,44,45,46,47].

Although each Y-family polymerase is recruited via a similar mechanism—by binding to monoubiquitinated PCNA—selection of the most appropriate polymerase is additionally influenced by (1) the precise DNA lesion, (2) the stabilization/degradation of the individual TLS polymerases, (3) additional protein-protein interactions with other TLS/replication proteins [48]. As we discuss in Section 5.1, this common means of TLS polymerase recruitment does, however, mean that the downregulation or mutation of the “correct” polymerase, or the upregulation of another TLS polymerase, can result in an “incorrect” polymerase being employed for lesion bypass. This can lead to an increase in the rate of mutagenesis within the cell.

While TLS is largely coordinated by PCNA monoubiquitination, TS is instead initiated following the further ubiquitination of these monoubiquitin moieties to form K63-linked ubiquitin chains [49,50] (Figure 4). This extension is mediated by the E2 and E2 variant enzymes Ubc13 and Mms2, in concert with one of two human orthologues of *Saccharomyces cerevisiae* Rad5, HLTF (helicase-like transcription factor), or SHPRH (SNF2 histone-linker PHD-finger RING-finger helicase) [51,52,53]. While it remains unclear how the switch from PCNA mono- to polyubiquitination is regulated, it is likely to involve the specific recruitment and activation of the Rad5 orthologues. For instance, in response to MMS-induced DNA damage, PCNA polyubiquitination seems to be dependent on SHPRH recruitment by TonEBP (tonicity-responsive enhancer-binding protein), a protein which itself binds to and encircles DNA following alkylation damage [54]. Recruitment of the Rad5 orthologues is, however, unlikely to be sufficient on its own for driving the switch to PCNA polyubiquitination, especially given findings that both of these proteins can also function in TLS to promote the binding of Y-family polymerases to monoubiquitinated PCNA. Indeed, SHPRH has been suggested to promote Pol κ recruitment following MMS-induced damage, while HLTF can promote recruitment of Pol η in response to UV exposure [55]. How exactly these paralogues are prompted to catalyze PCNA polyubiquitination, will therefore require further investigation.

Nevertheless, once formed, these chains create binding platforms for the ZRANB3 translocase, which directly associates with polyubiquitinated PCNA and promotes replication fork restart as well as suppression of unwanted sister chromatid exchange (SCE) following replication stress [56]. As with the role of PCNA monoubiquitination in TLS, the absolute requirement of PCNA polyubiquitination for activating TS has, however, also been called into question [57]. Such findings shed a different light on mammalian DNA damage bypass and could imply the subsequent involvement of other regulatory modifications. For instance, SUMO has been suggested to play a role in promoting TS in mammalian cells where PIAS1 and PIAS4 appear to mediate PCNA SUMOylation of residue K164 to preferentially promote TS [58].

### 4.2. PCNA Can Also Be Modified by SUMOylation

Several studies conducted in yeast have provided insight into the SUMOylation of PCNA involved in DNA damage bypass. SUMO-1 is preferentially conjugated to PCNA by the complex of E2/E3 enzymes, Ubc9, and Siz1 (PIASI in humans), at the same K164 residue as ubiquitin [59,60]. A second lysine residue K127 on yeast PCNA was also found to be a SUMO conjugation site, but only the K164 site of conjugation is highly conserved from yeast to humans [59]. The fact that ubiquitin and SUMO bind to the same site on PCNA leaves much speculation as to whether competition or collaboration exist between the two modifiers.

**Figure 5:**
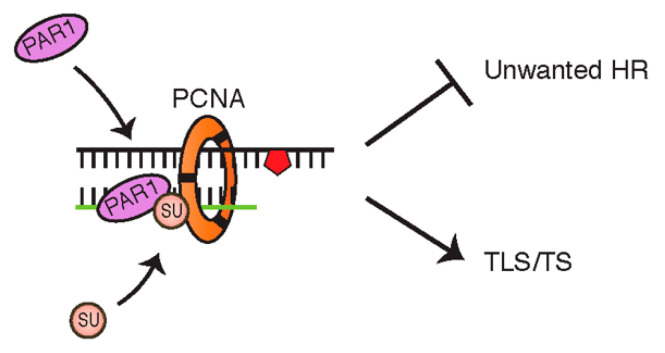
Schematic illustrating the binding of PCNA-associated recombination inhibitor (PAR1) to SUMOylated PCNA functioning to suppress unwanted recombination.

In the yeast model, the SUMOylation of PCNA is thought to stabilize the interaction between the helicase Srs2 and PCNA at the stalled replication fork, to suppress unwanted Rad51-dependent HR and divert toward a ubiquitin-dependent DNA damage response [61,62,63]. More specifically, it has been suggested that PCNA SUMOylation prevents formation of sister chromatid junctions produced by the Rad51 pathway, reinforcing its potentially impactful role in ensuring efficient functionality of Rad18/Rad5-dependent TLS/TS [64]. Only relatively recently was the SUMOylation of PCNA first observed in human cells, facilitating the enhanced binding of a potential Srs2 human homologue, PAR1 (PCNA-associated recombination inhibitor), and resulting in a similar response as in yeast, of preventing DSBs and unwarranted recombination (Figure 5) [65,66]. Although other functional human homologues of yeast Srs2 have been investigated—including FBH1 (F-box DNA helicase), RECQL5 (RECQ like helicase 5), and RTEL1 (regulator of telomere elongation helicase 1)—PAR1 is unique as it is the only one with a SUMO-interacting motif (SIM) [67,68,69,70].

### 4.3. PCNA Monoubiquitination Is Negatively Regulated by NEDDylation and ISGylation

**Figure 6:**
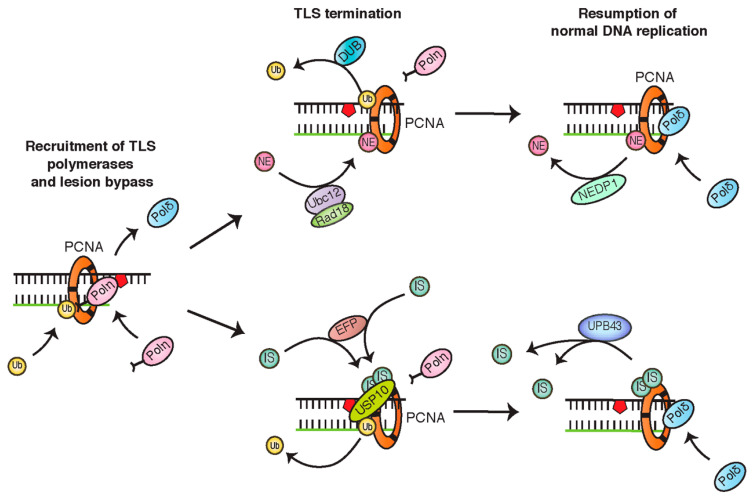
Schematics indicating the negative regulation of PCNA monoubiquitination by NEDDylation and ISGylation. Both modifications promote the deubiquitination of PCNA and translesion synthesis (TLS) termination.

Two other ubiquitin-like modifiers, NEDD8 and ISG15, are also thought to play a modulatory role in TLS. Recently, PCNA was found to be dynamically NEDDylated at K164 by the conjugating activity of Ubc12 and Rad18, in opposition to the deNEDDylating enzyme, NEDP1 [71]. Based on findings that enhanced PCNA NEDDylation increases cellular sensitivity to oxidative stress, it was proposed this modifier acts as an antagonist to ubiquitin, inhibiting Pol η recruitment. As levels of PCNA NEDDylation seem to increase following ubiquitination, this modification might serve to regulate the duration of TLS activity, in order to avoid additional risk of mutagenesis [71]. Similar to NEDDylation, ISGylation of PCNA in response to UV and other DNA damage-inducing agents has been suggested to function as a signal for timely TLS termination. In the proposed model, the monoubiquitination of PCNA recruits the E3 ligase, EFP, to conjugate ISG15 to PCNA at one of two sites, including the conserved K164 residue. The ISGylation of PCNA is then thought to recruit USP10 (ubiquitin-specific protease 10) to deubiquitinate PCNA, resulting in dissociation of Pol η and TLS completion [72] (Figure 6).

### 4.4. PCNA Monoubiquitination Is Opposed by Deubiquitinating Enzymes

In addition to USP10, other DUBs have been implicated in the regulation of PCNA monoubiquitination and TLS. USP1 is also responsible for deubiquitinating PCNA, and its degradation following UV exposure may play an important role in the subsequent accumulation of PCNA monoubiquitination [73]. Indeed, an increase in mutagenesis has been seen in both UV-treated and untreated USP1-depleted cells, supporting a role for this enzyme in the proper regulation of TLS [73]. USP7 is another key DUB that regulates multiple proteins involved in TLS, making it a versatile target candidate for cancer therapies. These include monoubiquitinated PCNA, as well as K48-linked polyubiquitinated Pol η [74] and Rad18 [75,76].

### 4.5. TLS Polymerases Are also Regulated by Ubiquitin and Ubiquitin-Like Proteins

In addition to binding ubiquitin, the TLS polymerases Pol η and Pol ι can themselves also be monoubiquitinated [45,77,78]. Interestingly, this seems to be dependent on the protein’s own ability to bind ubiquitin, as mutating the UBZ and UBM domains of Pol η and Pol ι, respectively, effectively inhibits these modifications [45]. One theory for why this might be is that the UBD domains of these proteins may allow them to interact with ubiquitin-charged E2 conjugating enzymes, to mediate a form of E3-independent ubiquitination. Indeed, the C-terminal half of Pol ι (containing UBM1 and UBM2) could be monoubiquitinated in vitro, when incubated with ubiquitin and an array of purified E2 enzymes [13]. This model has, however, been called into doubt by findings that Pol η interacts with and is monoubiquitinated by the E3 ligase, Pirh2 [79,80]. As Pirh2 depletion in these studies largely prevented Pol η monoubiquitination, it is unclear how these findings can be reconciled with an E3-independent mode of ubiquitination. The specific roles of the Pol η and Pol ι UBD domains in regulating monoubiquitination of these proteins may therefore need revisiting.

While Pol η is primarily monoubiquitinated at one of four C-terminal lysine residues [77,79], Pol ι contains a multitude of lysine residues dispersed along its length that can be monoubiquitinated in a mutually exclusive manner; a preference, however, exists for monoubiquitination of Pol ι C-terminal lysine residue K715 [78]. The monoubiquitination of Pol η seems to be inhibitory, as this modification occurs adjacent to the PIP box, sterically disrupting the proteins ability to interact with PCNA [77]. In addition, it has been suggested this modification might further disrupt Pol η function by mediating an intramolecular association with the Pol η UBZ domain. Consistent with such an inhibitory role, Pol η is deubiquitinated following cellular exposure to ultraviolet light [77]. By contrast, Pol ι monoubiquitination seems to be impervious to numerous forms of DNA damage [78]. An inhibitory role for this modification, however, also seems plausible, as monoubiquitination of K715 would likely be well-positioned to intramolecularly interact with the Pol ι UBM2 domain [78], reflecting the similar regulation of Pol η. Another possible explanation for the function of Pol ι monoubiquitination comes from findings that Pol η and Pol ι can form a ubiquitin-dependent complex [81], and that Pol ι expressed in frame with ubiquitin may be bound by Pol η [78]. The accuracy and compatibility of these models will, however, also require further study.

**Figure 7:**
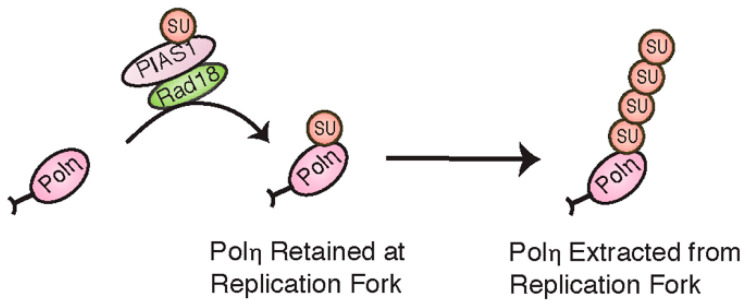
Mono- and polySUMOylation have opposing effects on Pol η function.

The ubiquitin-like modification, SUMO, has also recently been found to take part in regulating TLS polymerases. Conjugation of a single SUMO moiety to human Pol η at K163, thought to be mediated by Rad18 and PIAS1, has been suggested to help retain Pol η at replication forks in unchallenged cells [82] (Figure 7). Following DNA damage and the recruitment of Pol η to monoubiquitinated PCNA, PIAS1 is then thought to further SUMOylate Pol η to form polySUMO chains at multiple lysine residues. Unlike monoSUMOylation, these chains seem to be part of a negative feedback mechanism, marking Pol η for extraction from the replication fork by the SUMO-targeted ubiquitin ligase, RNF111 [83].

## 5. DNA Damage Bypass and Cancer

### 5.1. Deregulation of DNA Damage Bypass in Cancer

The proper regulation of DNA damage bypass is essential in preventing the accumulation of DNA mutations that may drive carcinogenesis. This is especially important given the relatively low fidelity of TLS polymerases, which if inappropriately employed, can mediate the misincorporation of dNTPs and potentially introduce somatic mutations. Indeed, while properly coordinated TLS can prevent mutagenesis by helping to ensure genomic duplication and by preventing replication fork collapse, imbalances that result in the use of an inappropriate TLS enzyme can substantially increase rates of nucleotide misincorporation. This is exemplified by the mutagenic participation of Pol ι and Pol κ in bypassing thymidine dimers in *Xeroderma Pigmentosum* Variant (XP-V) patients lacking a functional Pol η [28,29]. Consistent with this idea, the up- or down regulation of each of the Y-family DNA polymerases, as well as of Pol ζ, has been implicated in mutagenesis in multiple cancer types, presumably by inducing an imbalance that results in a TLS polymerase replicating past an adduct other than its cognate lesion [21,84]. Two cancer-related SNPs have also been detected in the UBM domains of Pol ι, representing one way through which ubiquitin-mediated regulation of TLS may be disrupted. While mutation of phenylalanine 507 to serine in UBM1 correlates with increased risk of prostate cancer development [85], mutation of threonine 706 to alanine in UBM2 has been observed with increased frequency in lung adenocarcinoma and squamous cell carcinomas [86].

Aside from the up/down regulation or mutation of individual TLS polymerases, deregulation of DNA damage bypass by ubiquitin and ubiquitin-like proteins may also play a significant role in driving carcinogenesis. This includes the altered expression of ubiquitin and ubiquitin-like proteins themselves; NEDD8 and ISG15, for example, are overexpressed in many cancers [87,88,89]. In addition, numerous ubiquitin and ubiquitin-like metabolism proteins directly associated with DNA damage bypass are deregulated in various cancers (Table 1). This includes Rad18, responsible for PCNA monoubiquitination, which is overexpressed in colorectal cancer, melanoma, and glioma cells [90]. This overexpression likely results in the increased monoubiquitination of PCNA, inappropriately activating TLS to drive cancer mutagenesis. Rad18 overexpression is commonly coupled with overexpression of its binding partner Melanoma Antigen-A4 (MAGE-A4) [91]. MAGE-A4 overexpression stabilizes Rad18 by protecting it from ubiquitin-mediated degradation, allowing for its continued over-activation in cancer cells [92].

Aside from Rad18, several other PCNA-interacting proteins have also been implicated in carcinogenesis. One such protein is ATAD5, which helps to unload PCNA from the DNA strand as well as to facilitate the deubiquitination of PCNA by interacting with USP1 [93]. Studies have designated ATAD5 as a key biomarker for ovarian cancer; deficient ATAD5 activity is linked to impaired PCNA unloading from the DNA strand and increased genomic instability [93]. SPRTN is another such protein involved in regulating the DNA damage response. SPRTN is believed to be recruited to ubiquitinated PCNA in order to prevent its deubiquitination by inhibiting USP1 activity (opposing the action of ATAD5) [93]. Mutations in SPRTN are often associated with development of hepatocellular carcinoma [94]. Another predominant E3 ligase frequently deregulated in cancer is HLTF [95]. As HLTF stimulates DNA damage bypass by template switching, the reduced activity of this protein instead likely functions to channel repair by translesion synthesis, again increasing the mutagenetic load of these cells [21].

### 5.2. Targeting DNA Damage Bypass in Cancer Therapies

While the deregulation of DNA damage bypass has an increasingly clear role in driving cancer mutations, once established, these as well as other replication stress pathways become critical for cancer cells to survive. This is due to the heightened levels of replication stress in cancer cells, resulting from increased oncogene expression that drives cell proliferation, while simultaneously disrupting cell cycle regulation and increasing the accumulation of reactive oxygen species [3]. Furthermore, cancer cells may rely on DNA damage bypass as a means of evading the replication stress caused by DNA-targeting anti-cancer agents. Pol η, for example, seems to be involved in bypassing lesions caused by platinum-based chemotherapeutics such as cisplatin [97,136]. Targeting DNA damage bypass may therefore prove to be a useful strategy in cancer therapy.

A number of promising therapeutic approaches have been developed in the past decade, that target the regulation of DNA damage bypass by ubiquitin and ubiquitin-like proteins. While compounds that target these processes have thus far only been tested in preclinical settings, many have demonstrated anti-cancer properties both in vitro and in vivo. T2AA (T2 amino alcohol), for instance, is a small molecule inhibitor that sensitizes cancer cells to cisplatin by binding to PCNA and interfering with TLS, resulting in DSB formation and the inability to replicate past inter-strand crosslinks (ICLs) [137]. PCNA can still be monoubiquitinated; however, T2AA binds to PCNA’s PIP-box binding cavity and disrupts the subsequent recruitment of Pol η and Rev1 during the TLS response to DNA damage [137]. Another class of PCNA inhibitors (PCNA-Is) has been discovered to bind directly to PCNA trimers thought to exist in the nucleoplasm and reduce their chromatin association, preventing them from being used during TLS [138]. Of the nine compounds, PCNA-I1 is the most potent and has been effective in retarding growth of human prostate tumor cells modeled in mice and upregulating the expression of Chk2 and p53 leading to apoptosis [139].

Logically, the opposite approach would be to target the TLS polymerases themselves to affect their PCNA-binding functionality in cancer cells. One study found that in both yeast and chicken DT40 cells, mutating the Rev1 UBM domains led to increased sensitivity to UV-light as well as other DNA-damaging agents, leading others to focus on Rev1 as a therapeutic target [47]. However, it is interesting to note that while both of Rev1′s UBM domains are essential for its association to damage-induced replication foci, only the UBM2 domain binds to ubiquitin in vitro [140]. Some promise has been seen in small molecule inhibitors, which bind to the Rev1 UBM2 domain and disrupt its association with monoubiquitinated PCNA. However, specifically targeting Rev1 seems to have more limitations in this case than directly targeting PCNA, as it calls for a greater degree of selectivity to distinguish the UBM domains of Rev1 from that of Pol ι [141].

Furthermore, Rev1 is significant because it acts as a scaffolding protein during TLS and is thought to mediate polymerase switching at stalled replication forks [27,142]. Compounds that bind to the C-terminal domain of Rev1 (Rev1-CT) interfere with its ability to interact with the Rev1-interacting regions (RIR) of other TLS polymerases and have been correlated with a decrease in the survival of human cancer cells when treated with UV-light or cisplatin [143]. A small molecule inhibitor, JH-RE-06, has recently been identified that binds to Rev1-CT and subsequently disrupts the Rev1-Rev7 (Pol ζ subunit) interaction by inducing Rev1 dimerization. JH-RE-06 is the first highly specific small molecule inhibitor that has been effective in obstructing mutagenic TLS and sensitizing tumors to chemotherapeutic treatments in vivo [144].

Another recent study has found that microRNA-145 (miR-145) can suppress expression of Rad18 in colorectal cancer (CRC) cells resulting in increased levels of DNA damage after 5-FU (5-fluorouracil) treatment [145]. Based on the observation that Rad18 is actually highly expressed in 5-FU-resistant CRC cells, miR-145 could play a significant role in debilitating the DNA damage response through its inhibitory effect on Rad18 and counteracting drug resistance in cancer cells [145]. Investigating methods to disrupt the function of DNA damage bypass proteins regulated by ubiquitin and ubiquitin-like modifiers in cancer cells could suppress their use of mutagenic TLS and make other chemotherapeutic agents more lethal and effective.

## 6. Conclusions

**Figure 8:**
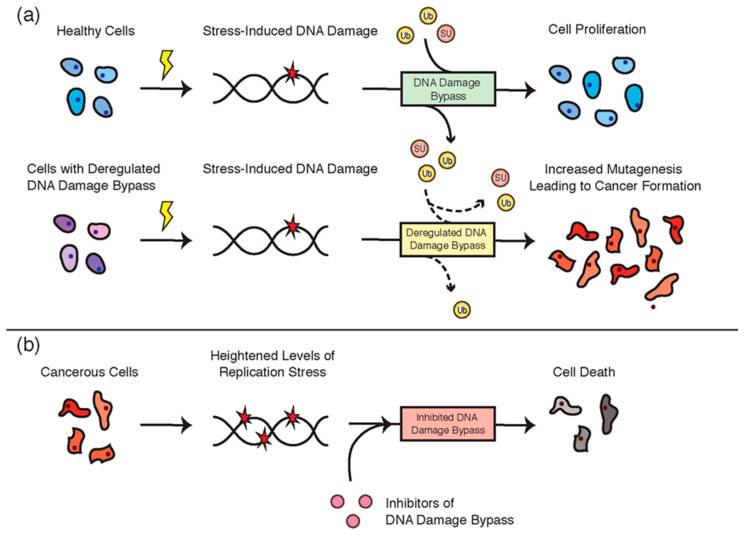
(**a**) The accurate regulation of DNA damage bypass by ubiquitin and ubiquitin-like proteins is essential for normal cellular function. Dashed lines indicate a deregulated flux of these modifiers. (**b**) Cancer cells experience heightened levels of cellular stress and rely on genome stability pathways such as DNA damage bypass for their survival. Targeting these pathways is a promising anti-cancer strategy.

The DNA damage bypass pathways are essential for ensuring the completion of replication in the presence of DNA damage. The accuracy of these pathways is, however, dependent on the strict regulation of the participant proteins, such as through post-translational modifications. In this review, we have discussed the central roles of ubiquitin and ubiquitin-like proteins in regulating TLS and TS. These modifiers are critical for preventing DNA mutagenesis and cancer development. Indeed, the deregulation of these processes is associated with the development of many different cancer types (Figure 8a,b). Ironically, however, while the deregulation of these pathways can drive cancer formation, DNA damage bypass is also an essential pathway used in many cancers to avoid replication stress. Targeting TLS and TS, such as through the disruption of ubiquitin and ubiquitin-like protein metabolism, is therefore a promising strategy for anti-cancer therapy. With the continued identification of roles that ubiquitin and ubiquitin-like proteins play in regulating DNA damage bypass, as well as the concurrent identification of regulatory enzymes that coordinate these modifications, the prospects for such therapies can only increase in coming years.

## Figures and Tables

**Table 1:** Many proteins that bind to and metabolize ubiquitin and ubiquitin-like proteins in the DNA damage response are deregulated or mutated in cancers.

Protein	Role in DNA Damage Response	Associated Cancers
Pol η	Y family TLS polymerase	Mutations/defects implicated in XP-V syndrome (high cancer susceptibility) [96] *Upregulated in*: bladder, non-small cell lung cancer, head and neck squamous cell carcinoma, ovarian cancer stem cells [97,98,99]
Pol ι	Y family TLS polymerase	*Upregulated in*: bladder cancer, breast cancer, basal cell carcinoma, esophageal squamous cell carcinoma, glioma (correlated with lymph node metastasis) [100,101,102,103,104] SNPs associated with prostate cancer, adenocarcinoma, squamous cell carcinoma [85,86]
Pol κ	Y family TLS polymerase	Mutations/defects implicated in prostate cancer, breast cancer [105,106] *Upregulated in*: glioma, non-small cell lung cancer [104,107]
Rev1	Y family TLS polymerase	*Upregulated in*: prostate cancer [108] SNPs associated with cervical squamous cell carcinoma [109]
PCNA	DNA sliding clamp	*Upregulated in*: prostate cancer, ovarian cancer (especially with lymph node metastasis) [110,111]
Rad18	E3 ubiquitin ligase	*Upregulated in*: colorectal cancer, primary and metastatic melanoma, glioma [90,112,113]
HLTF	E3 ubiquitin ligase	*Downregulated in*: colorectal, colon, stomach cancer, esophageal squamous cell carcinoma [114,115,116,117]
ATAD5	Unloading of PCNA from DNA strand	Mutations/defects associated with endometrial carcinoma [118] *Upregulated in*: epithelial ovarian carcinoma [119]
SPRTN	Stabilizing PCNA, resolving fork stalling DNA-protein crosslinks	Mutations/defects implicated in hepatocellular carcinoma [120]
USP1	Deubiquitinating enzyme	*Upregulated in*: cervical, stomach cancer, melanoma, sarcoma, osteosarcoma [121,122]
USP7	Deubiquitinating enzyme	*Upregulated in*: hepatocellular carcinoma, non-small cell lung cancer, epithelial ovarian cancer, myeloma [123,124,125,126]
USP10	Deubiquitinating enzyme	*Upregulated in*: prostate cancer, hepatocellular carcinoma [127,128]
NEDP1	De-NEDDylating enzyme	*Downregulated in*: hepatocellular carcinoma [129]
ISG15	Ubiquitin-like protein	*Upregulated in*: bladder, breast, endometrium, prostate cancer, hepatocellular carcinoma [130,131,132,133]
NEDD8	Ubiquitin-like protein	*Upregulated in*: hepatocellular carcinoma, nasopharyngeal carcinoma (correlated with lymph node metastasis) [134,135]
